# *Meripilus giganteus* ethanolic extract exhibits pro-apoptotic and anti-proliferative effects in leukemic cell lines

**DOI:** 10.1186/s12906-018-2366-7

**Published:** 2018-11-12

**Authors:** Monia Lenzi, Veronica Cocchi, Aleksandra Novaković, Maja Karaman, Marijana Sakač, Anamarija Mandić, Milica Pojić, Maria Cristina Barbalace, Cristina Angeloni, Patrizia Hrelia, Marco Malaguti, Silvana Hrelia

**Affiliations:** 1 Department of Pharmacy and Biotechnology, University of Bologna, Via San Donato 15, 40127 Bologna, Italy; 2 Institute of Food Technology, University of Novi Sad, Bul. Cara Lazara 1, Novi Sad, 21000 Serbia; 3 Faculty of Sciences, Department of Biology and Ecology, University of Novi Sad, Trg Dositeja Obradovića 2, Novi Sad, 21000 Serbia; 4 Department for Life Quality Studies, University of Bologna, Corso d’Augusto 237, 47921 Rimini, Italy; 5 School of Pharmacy, University of Camerino, Via Madonna delle Carceri, 9 - 62032 Camerino, MC Italy

**Keywords:** Meripilus giganteus, Cytotoxicity, Apoptosis, Chemoprevention, Flow-cytometry, Leukemic cell lines

## Abstract

**Background:**

The interest towards botanicals and plant extracts has strongly risen due to their numerous biological effects and ability to counteract chronic diseases development. Among these effects, chemoprevention which represents the possibility to counteract the cancerogenetic process is one of the most studied. The extracts of mushroom *Meripilus giganteus* (MG) (Phylum of Basidiomycota) showed to exert antimicrobic, antioxidant and antiproliferative effects. Therefore, since its effect in leukemic cell lines has not been previously evaluated, we studied its potential chemopreventive effect in Jurkat and HL-60 cell lines.

**Methods:**

MG ethanolic extract was characterized for its antioxidant activity and scavenging effect against different radical species. Moreover, its phenolic profile was evaluated by HPLC-MS-MS analyses. Flow cytometry (FCM) analyses of Jurkat and HL-60 cells treated with MG extract (0–750 μg/mL) for 24–72 h- allowed to evaluate its cytotoxicity, pro-apoptotic and anti-proliferative effect. To better characterize MG pro-apoptotic mechanism ROS intracellular level and the gene expression level of FAS, BAX and BCL2 were also evaluated. Moreover, to assess MG extract selectivity towards cancer cells, its cytotoxicity was also evaluated in human peripheral blood lymphocytes (PBL).

**Results:**

MG extract induced apoptosis in Jurkat and HL-60 cells in a dose- and time- dependent manner by increasing BAX/BCL2 ratio, reducing ROS intracellular level and inducing FAS gene expression level. In fact, reduced ROS level is known to be related to the activation of apoptosis in leukemic cells by the involvement of death receptors. MG extract also induced cell-cycle arrest in HL-60 cells. Moreover, IC_50_ at 24 h treatment resulted 2 times higher in PBL than in leukemic cell lines.

**Conclusions:**

Our data suggest that MG extract might be considered a promising and partially selective chemopreventive agent since it is able to modulate different mechanisms in transformed cells at concentrations lower than in non-transformed ones.

## Background

Mushrooms have been used for centuries as food all over the world, due to their unique taste and flavour [1]. Archaeological knowledge shows that humans have been using mushrooms since the Palaeolithic [2] and traditional uses in the treatment of infectious diseases have been previously described especially in Asian countries [3]. Over the last few years the interest in therapeutic potential of different species of lignicolous mushrooms has increased, justified by the traditional use of these organisms in the folk medicine of many countries [4, 5].

*Meripilus giganteus* (MG) is a ligniculous saprobiontic or parasite mushroom, which fructifies from summer to autumn at the base of broad-leaved trees, on stumps and roots, especially on beech wood. It derives its name from the remarkable dimensions that it is able to reach: up to a meter in diameter, protruding from the guest trunk for more than 30 cm, with a weight up to 10 kg. The upper portion is zoned, furrowed radially and concentrically by streaks of light brown to dark colour, wrinkled and covered with numerous scales. The tissue is initially soft and tenacious, and then becomes fibrous, leathery and whitish, blackening on contact or rubbing.

Although the young tops are edible after cooking, the completely grown mushroom is considered not edible due to its hard and tough consistency. For these reasons it is considered a species of little value in the culinary field.

Recently MG has drawn the attention of several scientists on its pharmacological properties such as antioxidant, antimicrobial, and anti-proliferative activities.

Karaman et al. [5, 6] investigated the antioxidant and antimicrobial activity of numerous lignicolous mushroom extracts. They demonstrated that MG extract exerts both DPPH radical (DPPH^**·**^**)** and hydroxyl radical (OH^**·**^) scavenging activity. Moreover, they demonstrated that the antioxidant activity of lignicolous mushroom extracts directly correlate with their phenolic content, that in MG are mainly represented by gallic and protocatechuic acids.

More recently, Maity et al. [7] isolated from the fruiting body of MG a polysaccharide (MGPS), which seems to possess an antioxidant capacity. In detail, it has been shown that increasing concentrations of MGPS are well correlated with the ability to scavenge OH^**·**^ and superoxide anion radical (O_2_^**·**-^). In order to have a more complete understanding of MGPS antioxidant mechanisms, the researchers also investigated its potential as a chelating agent of ferrous ions (Fe^2+^). Also in this case the ability of MGPS to chelate Fe^2+^ ions was demonstrated [7].

The results obtained from this study seem to confirm what was previously demonstrated by Rai et al. [8], who investigated the antioxidant properties of different MG extracts, finding a similar antiradical action against OH^**·**^ and O_2_^**·**-^.

Researchers investigated the antimicrobial potential of several fungal species, including MG, against five species of gram-positive bacteria, and four of gram-negative bacteria. The methanolic extracts of MG were shown to have a narrow spectrum of action against gram-negative bacteria, while strongly inhibit the growth of gram-positive species [6]. These data implement results previously obtained by Rai and co-workers [9], who described a moderate antibacterial action of MG against *E. coli* and *P. aeruginosa*.

Many substances with anti-proliferative effect have been isolated from fungi. Tomasi. et al. [10] analysed the effects of numerous methanolic mushroom extracts and demonstrated that MG exerts antiproliferative activity on 3LL murine lung cancer cell line. Previously Narbe et al. [11] isolated ergosterol peroxide from MG extract, which is known for its antiproliferative properties on both solid and liquid cancer models [12, 13].

## Aim of the study

Considering the numerous biological and pharmacological properties of MG extracts, it is possible to hypothesize its application as a chemopreventive agent in leukaemia. Therefore, the aim of this study was to evaluate the antitumor potential of MG ethanolic extract, fully characterized in its phenolic profile and antioxidant properties, in two different leukaemic cell lines. More specifically, its pro-apoptotic and anti-proliferative effects were analysed in human lymphoblastic leukaemia cells (Jurkat cells) and in human promyelocytic leukaemia cells (HL-60 cells). Moreover, expression level of genes involved in apoptotic pathways was evaluated. In addition to evaluating its selectivity towards cancer cells, its cytotoxic effect on non-transformed human peripheral blood lymphocytes (PBL) was also tested.

## Methods

### Materials

Sodium carbonate, Aluminium trichloride, Sodium acetate, Quercetin, Formic acid, FRAP reagent, Sodium nitroprusside (SNP), Griess reagent, TBA-reagent, Nitro Blue Tetrazolium (NBT), Ethylenediaminetetraacetic acid (EDTA), Ascorbic acid (AA), Gallic acid (GA), DPPH solution, Thiobarbituric acid (TBA), Trichloroacetic acid (TCA), Dimethyl sulfoxide (DMSO), Fetal Bovine Serum (FBS), Formaldehyde, Hanks’ balanced salt solution (HBSS), Histopaque-1077, Hoechst 33342, L-Glutamine (L-GLU), Penicillin-Streptomicin (PS), Phitohemagglutinin (PHA), Phosphate buffered saline (PBS), Primers (BAX, BCL2, FAS, GADPH, 18S rRNA), Roswell Park Memorial Institute (RPMI)1640 medium, Triton X-100, 2′-7′-dichlorodihydrofluorescin diacetate (DCFH-DA) were obtained from Sigma Co. (St. Louis, MO). Folin-Ciocalteu reagent (FC) was obtained from Merck (Darmstadt, Germany). Methanol (MeOH), Ethanol (EtOH) were purchased from Zorka (Šabac, Serbia). Guava Cell Cycle Reagent, Guava Nexin Reagent, Guava ViaCount Reagent (all from Merck, Darmstadt, Germany). RNeasy Mini Kit (from QIAGEN GmbH, Hilden, Germany), SsoAdvanced Universal SYBR Green Supermix, iScript cDNA Synthesis Kit (both from BIO-RAD, Hercules, CA, USA).

### *Meripilus giganteus* extract preparation

The extract was provided by the Institute of Food Technology (FINS) (Novi Sad, Republic of Serbia) as a part of the collaborative activities included in the Horizon 2020 project, FOODSTARS.

Mushrooms were collected in 2012 in the Sikole area (Serbia), fungal material was identified by Professor Maja Karaman (University of Novi Sad), expert in mycology. A voucher specimen of the fungal material has been deposited at “Buns herbarium” (Department of Biology and Ecology, University of Novi Sad, Serbia) with voucher number: 12–00697. After the exact determination of specie, mushrooms were stored at − 20 °C, freeze dried (Martin Christ GmbH, Germany) and ground to a fine powder. The extraction was obtained by macerating the powder (1 g) with 10 mL of 80% ethanol (EtOH) for 24 h in a shaker at room temperature (25 °C). The extract was filtered through Whatman No. 4 filter paper and, subsequently, the solvent was evaporated to dryness in a Rotavapor at 40 °C (Büchi, Switzerland) and stored. For further analysis the dried extract was dissolved in ethanol to obtain 5% (*w*/w) solution.

### Total phenolic content

Total phenolic content (TP) was determined in the ethanolic extract according to the method by Singleton et al. [14] and modified by Novaković et al. [15]. Briefly, 125 μL of Folin–Ciocalteu (0.1 M) reagent were added to 25 μL of the extract. After 10 min incubation, 100 μL of 7.5% sodium carbonate was added and the reaction mixture was incubated for 2 h. Absorbance was read at 690 nm in a plate reader (Multiskan Ascent, Thermo Electron Corporation). A standard curve was constructed using gallic acid in the range of 0 to 1000 μmol/L. Total phenolic content was expressed as mg gallic acid equivalents (GAE)/g of extract on dry weight basis.

### Total flavonoid content

The total flavonoid (TF) content of the ethanolic extract was determined by Chang et al. [16] modified for the measurements in a 96-well plate reader [15]. Briefly, 90 μL of methanol, 6 μL of aluminium trichloride (0.75 M), 6 μL of sodium acetate (1 M) and 170 μL of distilled water were added to 30 μL of the extract. After 30 min incubation absorbance was measured at 414 nm. A standard curve was constructed using quercetin in the range of 1.25–100 μg/mL. Results were expressed as mg quercetin equivalents (QE)/g of extract dry weight.

### HPLC–MS/MS determination of the phenolic compounds

Phenolic compounds were determined in the ethanolic extract according to the method of Orčić et al. [17], using an Agilent 1200 series liquid chromatograph equipped with a Zorbax Eclipse XDB-C18 RR 4.6 mm × 50 mm × 1.8 mm column (Agilent Technologies) at 40 °C. The separated compounds were detected by an Agilent series 6410A triple-quadrupole mass spectrometer with electrospray ionization (ESI). MassHunter ver. B.03.01. Software (Agilent Technologies) was used for instrument control and data analysis. The mobile phase consisted of 0.05% formic acid (A) and methanol (B) with a flow rate of 1 mL/min. The following gradient elution was used: at 0 min, 30% B, at 6.00 min reaching 70% B, then at 9.00 min 100% B, holding until 12.00 min, followed by equilibration time of 3 min to the starting mixture of 30% B. Samples were injected automatically, the injection volume for all samples was 5 μL. ESI parameters were: drying gas (N_2_) at 350 °C, flow of 9 L/min, nebulizer gas pressure of 40 psi, capillary voltage of 4 kV with negative polarity. All compounds were quantified in dynamic MRM mode (multiple reaction monitoring mode). The stock solution was prepared by mixing the solutions of 44 individual phenolic acids and flavonoids at concentration of 100 μg/mL each. Working standard solutions were prepared by dilution of stock solution in methanol–water (1:1, *v*/v) to obtain final concentrations in the range of 0.0015 to 25.0 μg/mL. Concentrations of compounds in the extract were determined from the peak areas using the equation for linear regression obtained from the calibration curves (*r*^*2*^ > 0.995) and expressed as μg/g dry weight.

### DPPH radical (DPPH^·^) scavenging activity

Free radical scavenging activity based on the monitoring of DPPH^·^ radical transformation in the presence of the ethanolic extract was determined as previously described by Espin et al. [18]. Briefly, the reaction mixture in the wells consisted of the extract (10 μL), DPPH solution (60 μL) and methanol (180 μL). The reaction mixture was incubated in the dark for 60 min at 25 °C. The absorbance was measured at 540 nm using a plate reader (Multiskan Ascent, Thermo Electron Corporation). Each sample was tested at five different concentrations in the range of 7.5–200 μg/mL to obtain IC_50._ The IC_50_ value (μg/mL) was defined as the concentration of an antioxidant extract which was required to quench 50% of the initial amount of DPPH· under the experimental conditions given.

### Ferric reducing antioxidant power (FRAP)

FRAP assay was performed on the ethanolic extract according to modified procedure of Benzie et al. [19]. The FRAP reagent is a mixture of 300 mM acetate buffer (pH 3.6): 10 mM 2,4,6-tris(2-pyridyl)-s-triazine (TPTZ) in 40 mM HCl: 20 mM FeCl_3_ (10:1:1, *v*/*v*/v). The reaction mixture in the wells consisted of the extract (10 μL), FRAP reagent (225 μL) and distilled water (22.5 μL). Absorbance was measured at 620 nm, after 6 min of incubation. Ascorbic acid was used to construct the standard curve and results were expressed as mg ascorbic acid equivalents (AAE)/g of extract on dry weight basis.

### Nitric oxide radical (NO^·^) scavenging capacity

NO scavenging capacity was determined according to the procedure of Green et al. [20]. Briefly, the reaction mixture in the test tubes consisted of the extract (30 μL), sodium nitroprusside (SNP) (500 μL) and 0.067 mol/L phosphate buffer at pH 7.4 (500 μL). Test tubes were incubated under light exposure at 25 °C for 90 min. After incubation, Griess reagent consisting of 0.2% solution of N-(1-naphthyl) ethylenediamine dihydrochloride (NEDA): 2% solution of sulphanilamide in 4% of phosphoric acid (1:1, v/v) was added (1 mL). Aliquots of the reaction mixture (250 μL) were transferred to the plate, and their absorbances were measured using a plate reader at 540 nm (Multiskan Ascent, Thermo Electron Corporation). Samples were tested at five different concentrations in the range of 74–591 μg/mL to obtain IC_25_, defined as the concentration of the extract which scavenges 25% of the initial amounts of NO^.^.

### Hydroxyl radical (OH^·^) scavenging capacity

Determination of scavenging activity on OH^**·**^, generated in Fenton reaction, was performed from the reaction of 2-deoxyribose degradation [21]. The reaction mixture contained 100 μL of 2-deoxyribose, 100 μL of FeSO_4_ (127 mg FeSO_4_·7 H_2_O in 50 mL of phosphate buffer, pH 7.4) and 10 μL of the tested extract. Phosphate buffer was added to the mixture to the final volume of 3 mL and incubated at 37 °C for 1 h. Two millilitres of TBA-reagent (10.4 mL of 10% HClO_4_, 3 g of thiobarbituric acid (TBA) and 120 g of 20% trichloroacetic acid (TCA) dissolved in 800 mL of distilled H_2_O) and 0.2 mL of 0.1 M EDTA were added to terminate the reaction. The absorbance was measured at 532 nm using a UV-Vis spectrophotometer (Agilent Technologies, Santa Clara, CA). To obtain IC_50_ value, the range of concentrations of 0.7–4.7 μg/mL MG extract was tested.

### Superoxide anion radical (O_2_^-.^) scavenging activity

Superoxide anion radical scavenging activity of the ethanolic extract was determined by measuring its ability to neutralize superoxide anion radicals generated during aerobic reduction of nitro blue tetrazolium (NBT) by NADH, mediated by 5-methylphenazin-5-ium methyl sulphate (PMS) [22]. The reaction mixture in a test tube was composed by 677 μM NADH (100 μL), 60 μM PMS (100 μL), 144 μM NBT (200 μL), 0.017 mol/L phosphate buffer at pH 8.3 (1,1 mL) and the extract (10 μL). After 5 min of incubation, aliquots (250 μL) were transferred to the plate wells (Multiskan Ascent, Thermo Electron Corporation), and their absorbances were measured at 540 nm. Five different concentrations of each sample in the range of 2.4–33.1 μg/mL were tested to obtain IC_50_, defined as the concentration of the extract able to scavenge 50% of O_2_^**·**-^.

### Jurkat and HL-60 cell culture and treatments

Jurkat cells (acute T-cell lymphoblastic leukaemia) and HL-60 cells (acute promyelocytic leukaemia) were purchased at the “Istituto Zooprofilattico” of Lombardia and Emilia-Romagna (Brescia, Italy). Both cell lines were cultured at 37 °C and 5% CO_2_ in Roswell Park Memorial Institute (RPMI)1640 medium supplemented with 1% Penicillin-Streptomicin (PS), 1% L-Glutamine (L-GLU) and 10% of Fetal Bovine Serum (FBS) for Jurkat cells and 20% of FBS for HL-60 cells (all from Sigma Aldrich, Saint Luis, MO, USA).

The MG extract was dissolved in RPMI at 20% of DMSO (*v*/v), in order to obtain a Working Solution 50 mg/mL. The solution thus prepared has been stored for a maximum of 72 h at − 20 °C and protected from light. The concentrations of the different extracts tested ranged from 0 to 750 μg/mL and the concentration of DMSO was always within the 0.05–1% range in all experimental conditions.

In particular, 3.75 × 10^5^ of Jurkat cells were treated with increasing concentrations of extract from 0 to 500 μg/mL and incubated for 24, 48 and 72 h. The cell density never exceeded the critical value of 3.00 × 10^6^ cells/mL of medium. One hundred twenty-five thousand of HL-60 cells were treated with increasing concentrations of extracts from 0 to 750 μg/mL and incubated for 24, 48 and 72 h. The cell density never exceeded the critical value of 1.00 × 10^6^ cells/mL of medium.

### PBL culture and treatments

Authorization to the use of human blood samples (Buffy coat), for research purposes, was obtained from AUSL of Bologna, Italy, S. Orsola-Malpighi Hospital -PROT GEN No. 0051937, and written informed consent was obtained by AUSL of Bologna, Italy, S. Orsola-Malpighi Hospital from donors for the use of their blood for scientific research purposes. PBL were isolated from the whole peripheral blood of 5 AVIS healthy donors (Association of Italian Blood Volunteers), by density gradient centrifugation with Histopaque-1077 (Sigma Aldrich, Saint Luis, MO, USA) [23].

PBL were cultured at 37 °C and 5% CO_2_ in RPMI-1640 supplemented with 1% PS, 1% L-GLU, 15% FBS and in the presence of 0.5% Phitohemagglutinin (PHA) (Sigma Aldrich, Saint Louis, MO, USA) for 48 h to stimulate cell proliferation. Two hundred thousand PBL were than treated with increasing concentrations of extract from 0 to 1000 μg/mL and incubated for 24 h.

### Flow cytometry (FCM)

All FCM analyzes reported below were performed using a Guava easyCyte 5HT flow cytometer equipped with a class IIIb laser operating at 488 nm (Merck, Darmstadt, Germany).

### Cytotoxicity analysis by FCM

The cytotoxicity induced by MG was evaluated by the *Guava ViaCount Assay protocol.* In particular, the percentage of viable cells was assessed by FMC using the Guava ViaCount Reagent (Merck, Darmstadt, Germany) that contains the dye Propidium Iodide (PI) and analyzed by Guava ViaCount software [24].

The results obtained in the samples treated with different concentrations of extracts were normalized on those obtained in control cultures and used to calculate the IC_50_ by interpolation from the dose-response curve. In the subsequent experiments concentrations ≤ IC_50_ were used.

### Analysis of apoptosis by FCM and optical microscopy

The discrimination of the death mechanism was assessed by the *Guava Nexin Assay Protocol.* In particular, the percentage of live, apoptotic and necrotic cells was assessed by FCM using the Guava Nexin Reagent (Merck, Darmstadt, Germany) that containing 7-aminoactinomycin D (7-AAD) and Annexin-V-PE and analyzed by Guava Nexin software as previously reported [24, 25].

Moreover, the nuclear condensation and fragmentation associated with the apoptotic process was evaluated by fluorescence microscopy with 100X magnification. After treatment, 1.00 × 10^6^ cells were loaded into cytospin chamber and centrifuged ad 450 rpm for 10 min. Cells were than fixed in formaldehyde 3.7%, washed in PBS pH 7.2, permeabilized in 0.15% triton X-100 (all Sigma-Aldrich, Saint Louis, MO, USA) and nuclei were stained using Hoechst 33342 500 nM as previously reported [26].

### Cell-cycle analysis by FCM

The effect of MG on the cancer cells replication was evaluated by *GUAVA Cell-Cycle Assay protocol.* In particular, the percentage of cells in the different phases of the cell-cycle (G_0_/G_1_, S, G_2_/M) was assessed by FCM using the Cell-Cycle Reagent (Merck, Darmstadt, Germany) that containing PI and analysed by Guava Cell-Cycle software [24].

### RNA extraction

After 16 h treatments, total RNA was extracted from Jurkat and HL-60 cells by using RNeasy Mini Kit (QIAGEN GmbH, Hilden, Germany), following the manufacturer’s protocol. The yield and purity of the RNA were measured using NanoVue Spectrophotometer (GE Healthcare, Milan, Italy).

### Analysis of FAS, BAX and BCL2 mRNA expression levels by reverse transcriptase polymerase chain reaction

cDNA was obtained by reverse transcribing mRNA starting from 1 μg of total RNA using iScript cDNA Synthesis Kit (BIO-RAD, Hercules, CA, USA), following the manufacturer’s protocol. The subsequent polymerase chain reaction (PCR) was performed in a total volume of 10 μl containing 2.5 μl (12.5 ng) of cDNA, 5 μl SsoAdvanced Universal SYBR Green Supermix (BIO-RAD, Hercules, CA, USA) and 0.5 μl (500 nM) of each primer. The primers used are reported in Table 1 18S rRNA and GAPDH were used as reference genes.

**Table 1:** Both forward and reverse primer sequences for BAX, BCL2, FAS, GAPDH and 18S rRNA genes are reported

Gene	5′-Forward-3′	5′-Reverse-3′
BAX	AACTGGACAGTAACATGGAG	TTGCTGGCAAAGTAGAAAAG
BCL2	GATTGTGGCCTTCTTTGAG	GTTCCACAAAGGCATCC
FAS	CTGTCCTCCAGGTGAAAG	TGTACTCCTTCCCTTCTTG
GAPDH	ACAGTTGCCATGTAGACC	TTGAGCACAGGGTACTTTA
18S rRNA	CAGAAGGATGTAAAGGATGG	TATTTCTTCTTGGACACACC

### Intracellular ROS level

After 24 h treatment Jurkat and HL-60 cells (1.00 × 10^6^ cells/mL), were washed twice in HBSS, and incubated with 5 μM 2′-7′-dichlorodihydrofluorescin diacetate (DCFH-DA) (Sigma-Aldrich, Saint Louis, MO, USA), for 20 min at 37 °C. When inside the cell, DCFH-DA is deacetylated and can be oxidized by ROS to the highly fluorescent 2′,7′-dichlorofluorescein (DCF). DCF fluorescence was measured using a multi-well plate reader (Wallac Victor2, PerkinElmer) at excitation and emission wavelengths of 485 and 535 nm, respectively [27]. Fluorescence values were reported as percentage of intracellular ROS with respect to control.

### Statistical analysis

Results on TP and TF content, on DPPH^**·**^, OH^**·**^, O_2_^**·**-^, NO^**·**^ scavenging activity and FRAP are expressed as mean ± standard deviation (SD). All results on cell viability, analysis of apoptosis, cell-cycle, gene expression and intracellular ROS are expressed as mean ± standard error mean (SEM) of at least five independent experiments. For the statistical analysis of apoptosis, cell-cycle and gene expression level we used the Analysis of Variance for paired data (repeated ANOVA), followed by Bonferroni as the post-test. For statistical analyses of ROS intracellular level we used the t-test for paired data. All the statistical analyses were performed using GraphPad Software Prism 6 (GraphPad Software LLC, La Jolla, CA, USA).

## Results

### MG phenolic composition

TP content of MG ethanolic extract resulted in 106.33 ± 11.27 mg GAE/g extract, while TF content resulted in 13.84 ± 1.11 mg QE/g extract. HPLC-MS/MS analyses revealed the presence of *p*-OH-benzoic acid, protocatechuic acid, *p*-coumaric acid and caffeic acid. Quantitative determination is reported in Table 2.

**Table 2:** Content of phenolic compounds in MG extract

Phenolic compounds	μg/g dry weight
p-OH-benzoic acid	23.9 ± 3.66
Protocatechuic acid	1.26 ± 0.13
p-coumaric acid	0.2 ± 0.05
Caffeic acid	1.3 ± 0.11

Values are expressed as means ± SD of triplicate measurements. Phenolic acid contents were determined by HPLC-MS/MS as described in the Methods section

### Antioxidant activity of MG extract

Since different mechanisms are involved in the neutralization of different radical species Table 3 reports the antioxidant activity of MG extract measured according to five methods, DPPH^.^, NO^.^, OH^·^ and O_2_^·-^ scavenging capacity and FRAP.

**Table 3:** Antioxidant activity of MG extract

DPPH^.^ (IC_50_)^a^ μg/mL	57.77 ± 2.48
NO^.^ (IC_25_)^b^ μg/mL	148.04 ± 4.98
OH^·^ (IC_50_)^a^ μg/mL	1.74 ± 0.2
O_2_^−^ (IC_50_)^a^ μg/mL	24.28 ± 2.06
FRAP^c^ mg (AAE)/g	22.54 ± 3.51

Values are expressed as means ± SD of triplicate measurements. Antioxidant activity was determined as described in the Methods section

^a^IC_50_ (μg/mL), concentration of extract that neutralized 50% of DPPH·, OH· and O_2_^.-^

^b^IC_25_ (μg/mL), concentration of extracts that neutralized 25% of NO

^c^Ferric reducing antioxidant power (FRAP) is expressed as mg ascorbic acid equivalents/g extract dry weight (mg AAE/g d.w)

### Cytotoxicity analysis on Jurkat and HL-60 cells

**Fig. 1:**
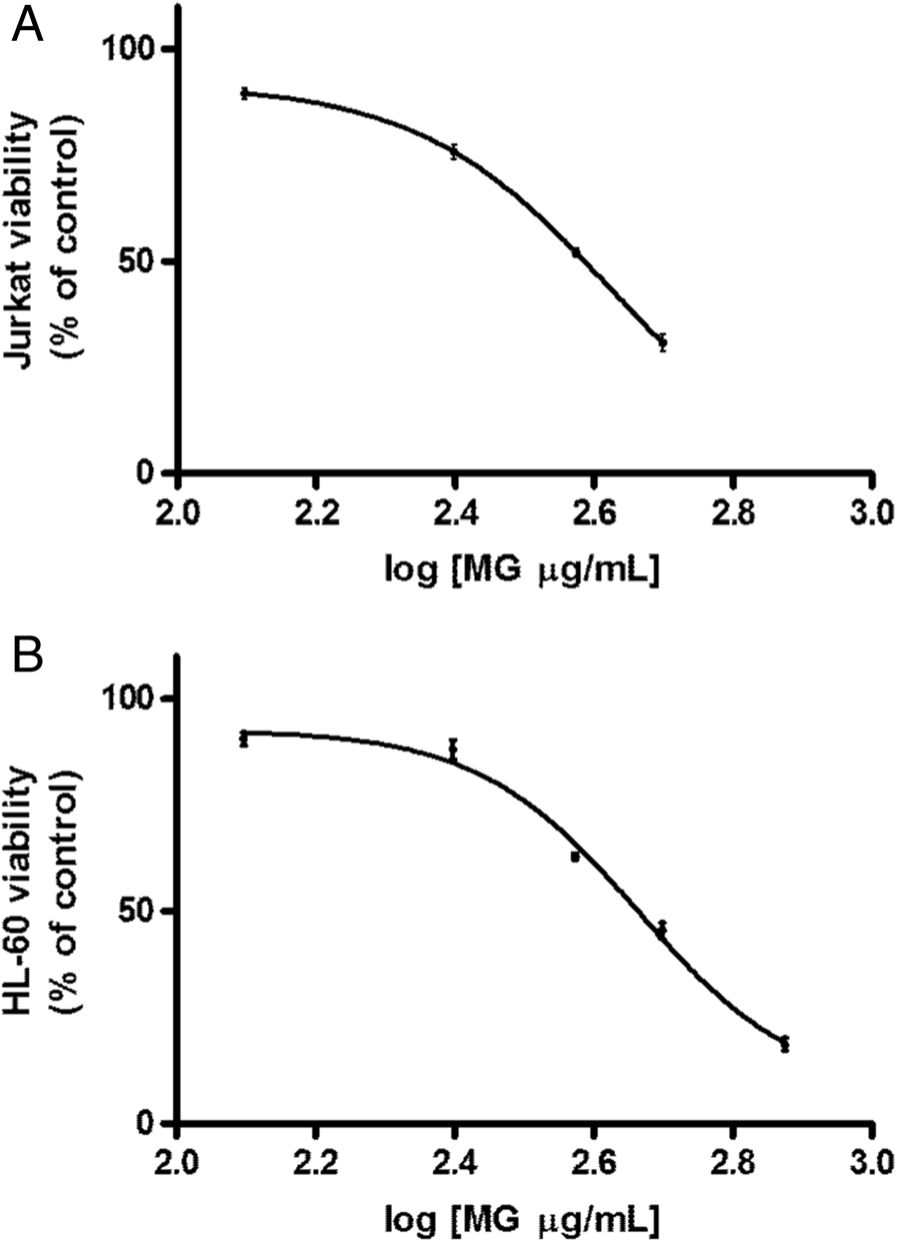
Effect of MG on viability of Jurkat and HL-60 cells. Cell viability was determined as described in Methods section. IC_50_ obtained by curve fitting of viable cells after 24 h treatment with MG for Jurkat cells (**a**) and HL-60 cells (**b**). Data are presented as means ± SEM of five independent experiments

After 24 h treatment the IC_50_ for MG was found to be 385 μg/mL in Jurkat cells and equal to 461 μg/mL in HL-60 (Fig. 1).

### Apoptosis analysis on Jurkat and HL-60 cells

In order to evaluate the involvement of a specific cell death mechanism in the demonstrated cytotoxic action, the analysis of apoptosis possibly induced at different concentrations on Jurkat and HL-60 cells was performed, after 24, 48 and 72 h, in order to determine if the event was dose and/or time-dependent.

Specifically, the cells were treated with concentrations ≤ IC_50_ and the double staining Annexin V-PE / 7-AAD allowed to measure the percentage of live, apoptotic and necrotic cells.

**Fig. 2:**
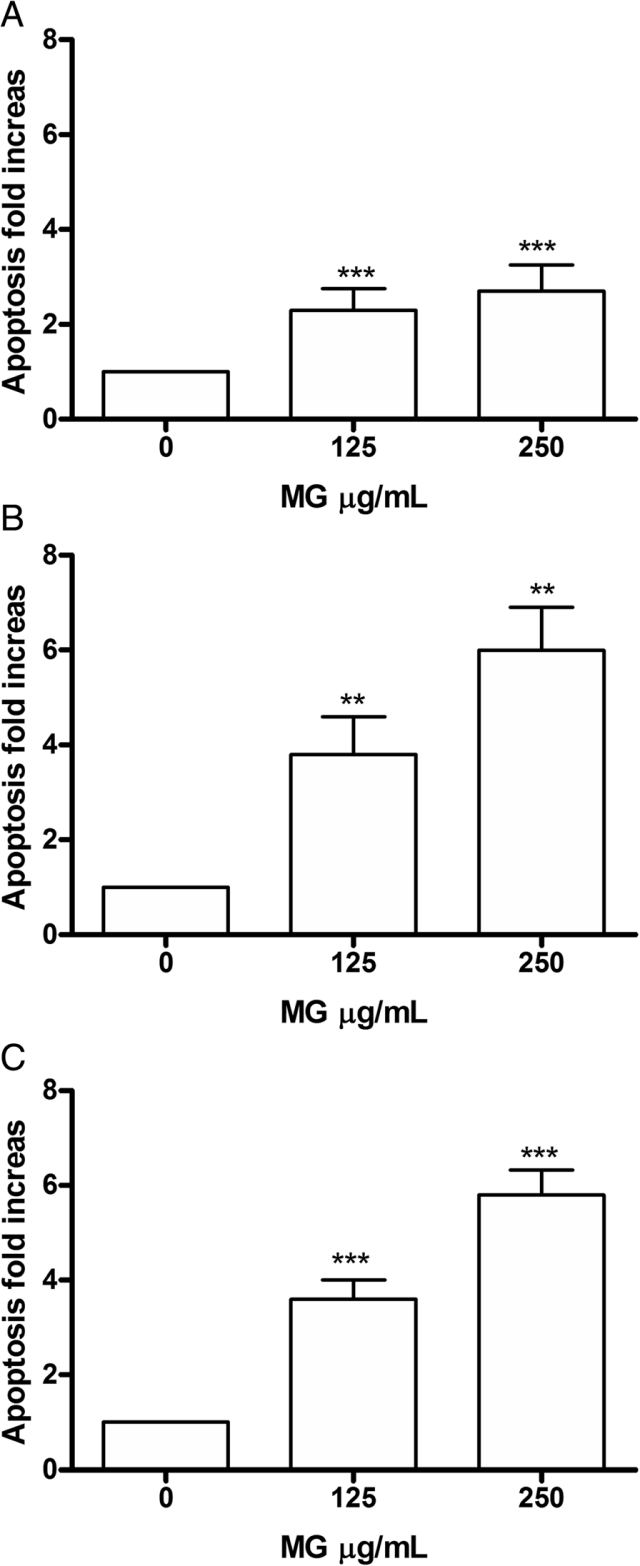
Increase in apoptotic Jurkat cell fraction after MG treatment. Apoptosis was evaluated at 24 h (**a**), 48 h (**b**) and 72 h (**c**), as reported in Methods section. Each bar represents means ± SEM of five independent experiments. Data were analysed using repeated ANOVA followed by Bonferroni post-test.^**^p < 0.01 vs control; ^***^p < 0.001 vs control

In Jurkat cells, MG showed a dose- and time-dependent induction of apoptosis. In fact after 24 h there was a statistically significant increase of apoptotic cells percentage at the concentration 125 μg/mL equal to 2 times compared to the control (7.3% vs 3.1%) and an increase equal to 3 times at 250 μg/mL (8.4% vs 3.1%). After 48 h, on the other hand, a doubling of the fraction of apoptotic cells compared to 24 h was observed at both concentrations tested. Specifically there was a 4 times increase at 125 μg/mL (13.3% vs 3.5%) and a 6 times increase at 250 μg/mL (21.2% vs 3.5%). At 72 h the trend is comparable (Fig. 2a, b, c).

**Fig. 3:**
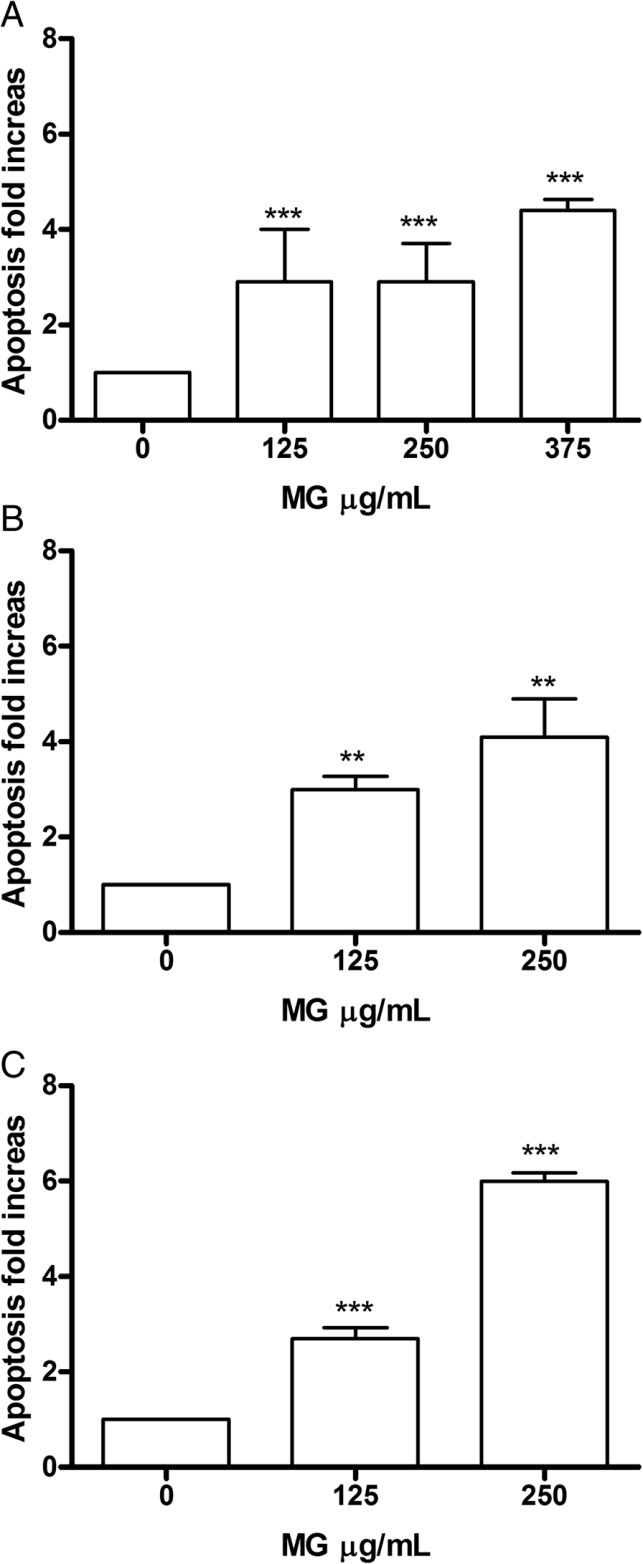
Increase in apoptotic Jurkat cell fraction after MG treatment. Apoptosis was evaluated at 24 h (**a**), 48 h (**b**) and 72 h (**c**), as reported in Methods section. Each bar represents the mean ± SEM of five independent experiments. Data were analysed using repeated ANOVA followed by Bonferroni post-test. ^**^p < 0.01 vs control; ^***^p < 0.001 vs control

In HL-60 (Fig. 3) after 24 h there was evidence at the concentration of 125 μg/mL, a statistically significant increase in the fraction of apoptotic cells, equal to 3 times compared to control (11.3% vs. 3.8%), parallel to an increase of 3 and 4 times, for 250 μg/mL and 375 μg/mL, respectively (11.1% vs. 3.8, 16.2% vs 3.8%). After 48 h, the trend remained comparable to 24 h for the 125 μg/mL concentration (11.6% vs 3.7%), while for 250 μg/mL a further increase in the number of apoptotic cells was observed, equal to at 4 times the control (15.2% vs. 3.7%); the same concentration, at the longest treatment time (72 h), was instead associated with an increase equal to 6 times (8.6% vs 1.4%). Therefore, these data demonstrate a dose- and time-correlated trend at all times analysed, particularly evident for the concentration of 250 μg/mL.

**Fig. 4:**
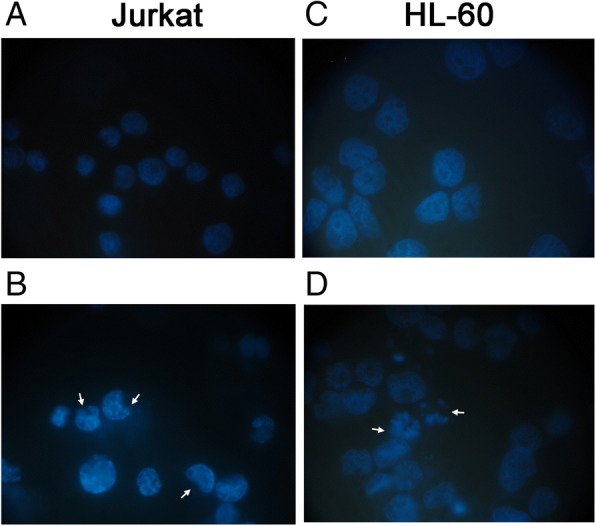
Fluorescence microscopy analysis of Jurkat and HL-60 after MG treatment. Nuclear condensation and fragmentation associated to apoptotic process on Jurkat and HL-60 cells was evaluated by fluorescence microscopy at 100x magnification after 24 h of 250 μg/mL MG treatment (**b**, **d**) respect to control culture (**a**, **c**). White arrows indicate condensed and/or fragmented nuclei as marker of apoptosis

Considering the demonstrated pro-apoptotic effect, we wanted to confirm the results obtained by FCM, also by fluorescence microscopy analysis, visualizing the morphological changes characteristic of apoptosis, such as nuclear condensation and fragmentation (Fig. 4).

### Cell-cycle analysis on Jurkat and HL-60 cells

**Fig. 5:**
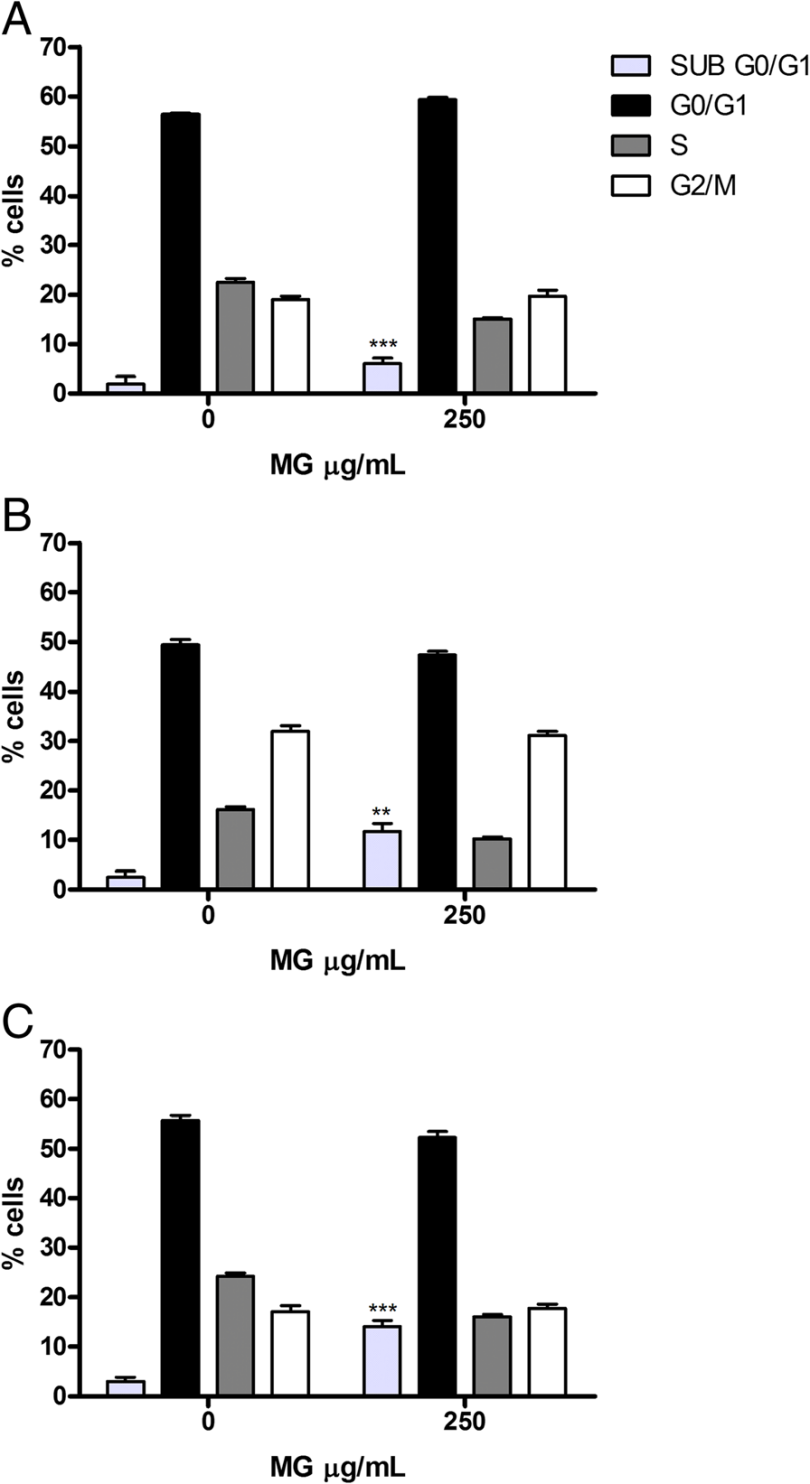
Cell-cycle analysis of Jurkat cells treated with MG extract. Fraction of Jurkat cells in the different phases of the cell cycle after MG treatment for 24 h (**a**) 48 h (**b**) and 72 h (**c**) was evaluated as reported in Methods section. Each bar represents the mean ± SEM of five independent experiments. Data were analysed using repeated ANOVA followed by Bonferroni post-test and revealed no statistically significant differences

In order to evaluate whether the induction of apoptosis caused by MG was an independent event or subsequent a slowing/blocking of the cell-cycle, the Jurkat and HL-60 cells were treated with the concentration selected on the basis of the results obtained from the apoptosis analysis and incubated at the same treatment times. The PI staining allowed to highlight that MG does not show any activity on the cell-cycle of Jurkat cells, at the tested concentration (250 μg/mL), at any treatment time (Fig. 5a, b, c).

**Fig. 6:**
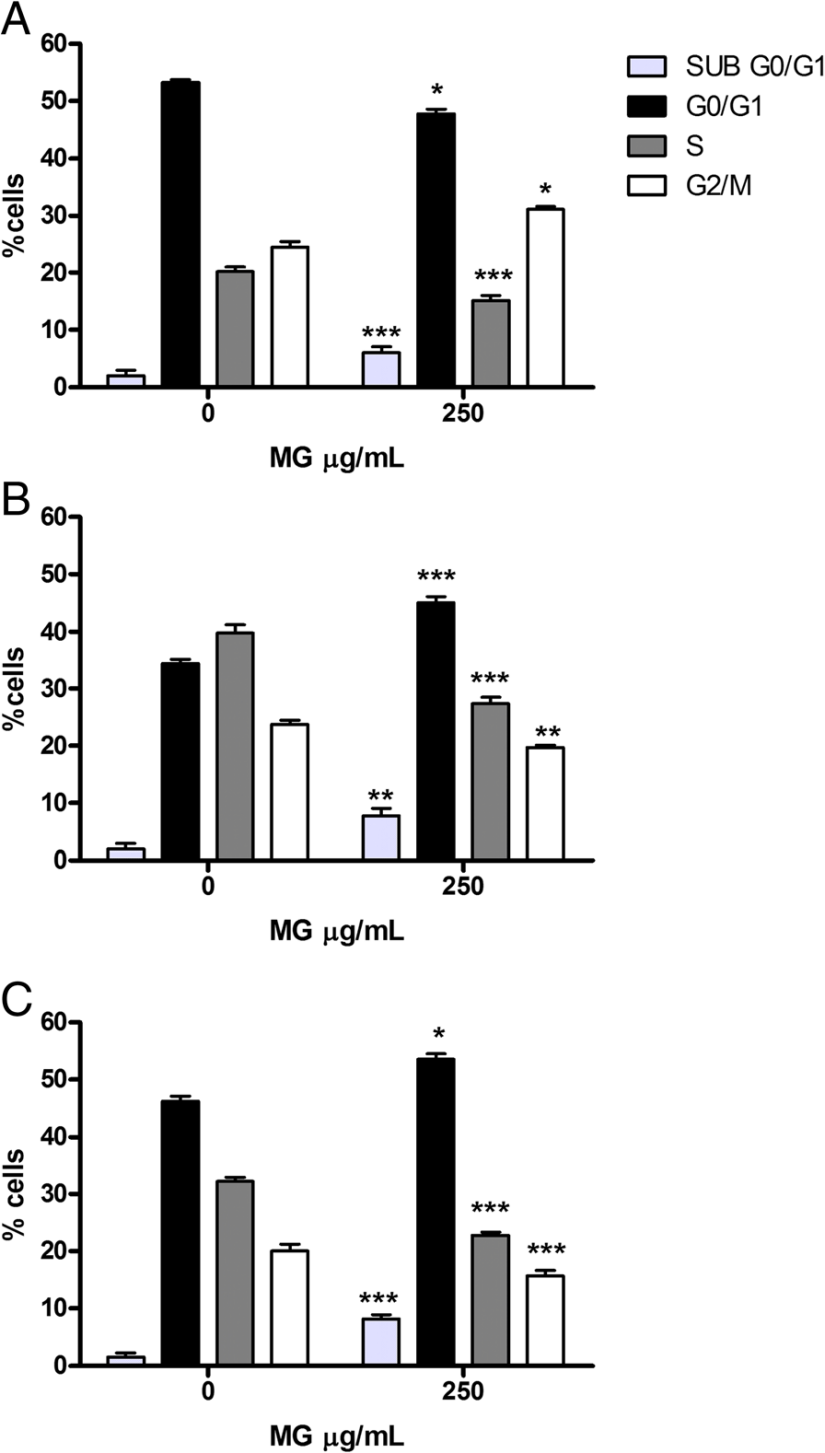
Cell-cycle analysis of HL-60 cells treated with MG extract. Fraction of HL-60 cells in the different phases of the cell-cycle after MG treatment for 24 h (**a**) 48 h (**b**) and 72 h (**c**) was evaluated as reported in Materials and Methods section. Each bar represents the mean ± SEM of five independent experiments. Data were analysed using repeated ANOVA followed by Bonferroni post-test. ^*^p < 0.05 vs control; ^**^p < 0.01 vs control; ^***^p < 0.001 vs control

Conversely, on HL-60 cells, after 24 h a slowing of the cell-cycle is observed in the G_2_/M phase, underlined by an increase in the cell fraction equal to a 33.1% vs 25.2% in the control cultures. At 48 h instead, there was an increase in the number of cells in the G_0_/G_1_ phase (47.7% vs 35.1%), with a consequent reduction in S phases (30.0% vs 40.4%) and G_2_/M (21.8% vs 24.5%); the same behaviour is observed at 72 h (Fig. 6a, b, c).

### FAS, BAX and BCL2 expression analysis on Jurkat and HL-60 cells

mRNA expression analysis of three genes involved in apoptotic pathways regulation such as FAS, BAX, BCL2 were performed.

**Fig. 7:**
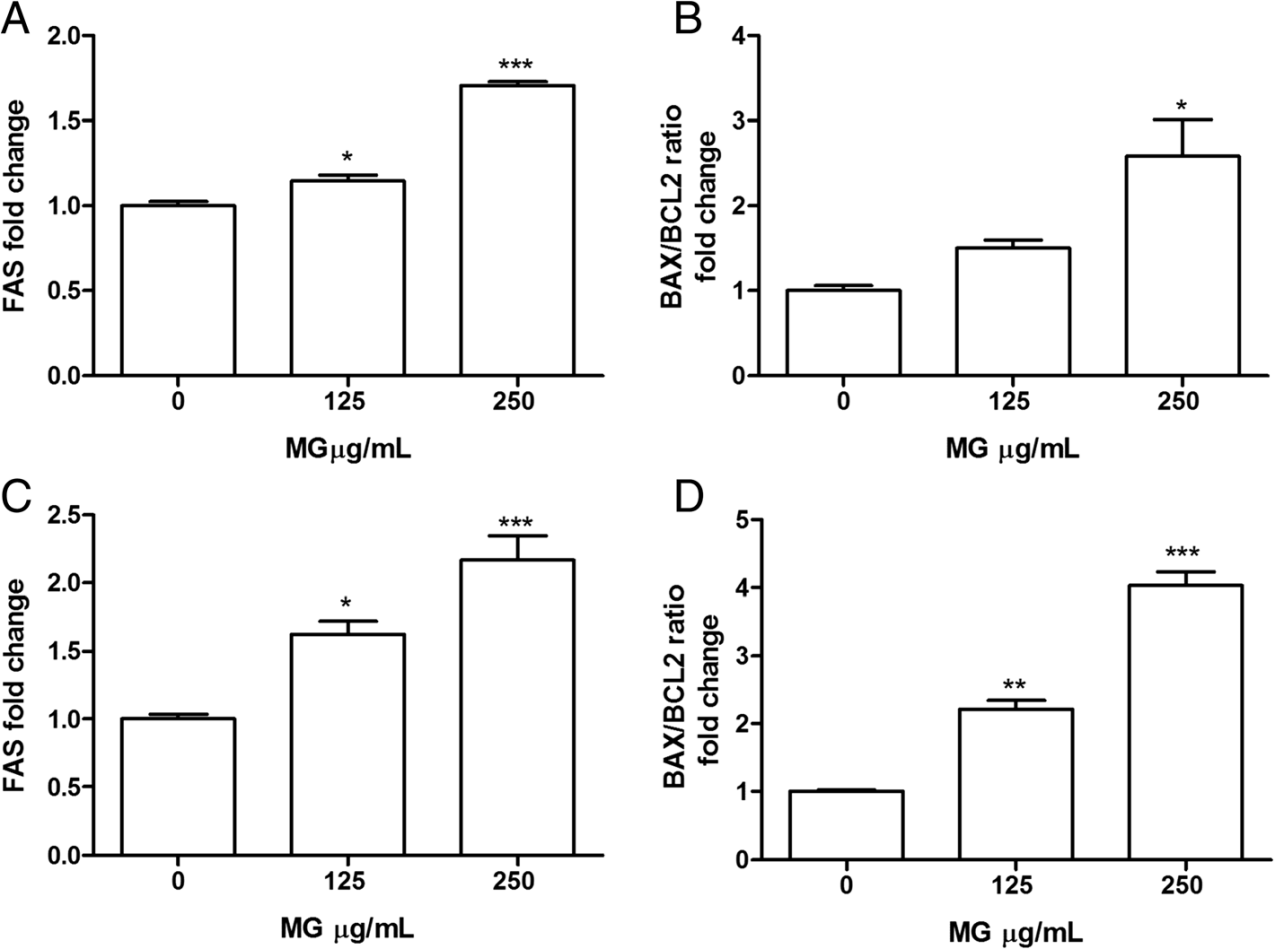
Effect of MG extract treatment on FAS, BAX and BCL2 expression level in Jurkat (**a**, **b**) and HL-60 (**c**, **d**) cells. Total RNA was isolated, and the mRNA level of target genes was quantified using RT-PCR normalized to 18S rRNA and GAPDH as reference genes. Triplicate reactions were performed for each experiment. Each bar represents the mean ± SEM of three independent experiments. Data were analysed by one-way ANOVA followed by Bonferroni’s test. ^*^p < 0.05 vs control; ^**^p < 0.01 vs control;^***^p < 0.001 vs control

Figure 7 shows that MG extract after 16 h treatment significantly induced, in both Jurkat and HL-60, FAS mRNA expression level (Fig. 7a, c) and increases the ratio between BAX and BCL2 mRNA expression (Fig. 7b, d).

### Intracellular ROS level

**Fig. 8:**
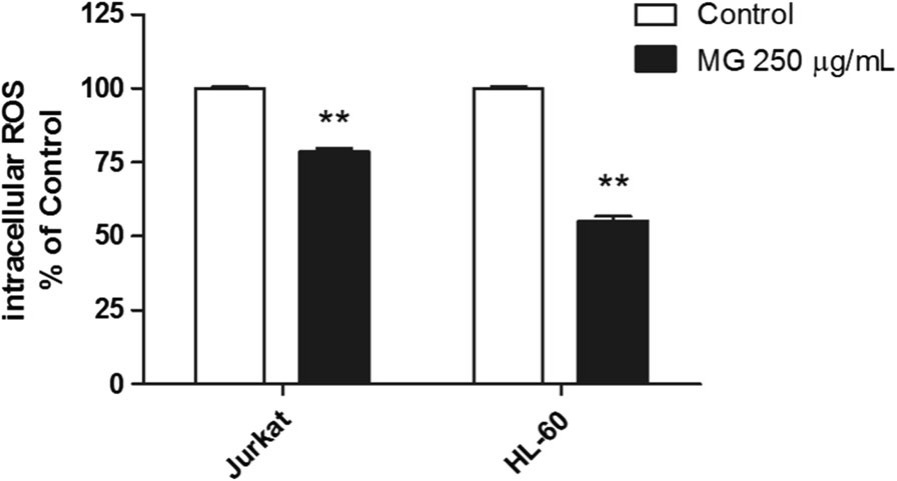
Intracellular ROS level in Jurkat and HL-60 cells treated with MG at 250 μg/mL for 24 h. Each bar represents the mean ± SEM of three independent experiments. Data were analysed by t-test, ^**^p < 0.01 vs control

To characterize the mechanism behind MG capability to induce apoptosis in leukaemia cells its potential modulatory effect on intracellular ROS level was investigated in both Jurkat and HL-60 cells by the DCFH-DA assay. As shown in Fig. 8, ROS intracellular level was significantly decreased respect to control by MG 250 μg/mL after 24 h treatment in both cell-lines.

### Cytotoxicity analysis on PBL

**Fig. 9:**
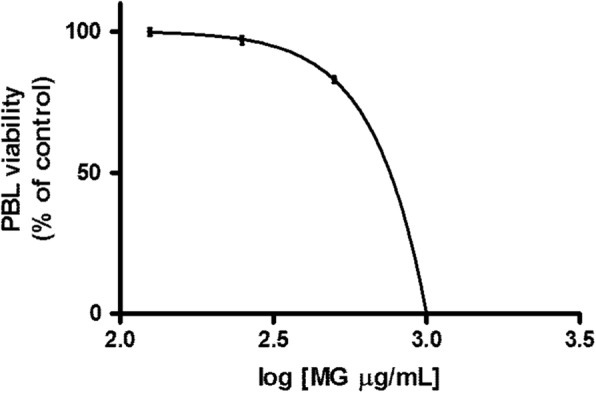
Effect of MG on viability of PBL. IC_50_ was obtained by curve fitting of viable cells after 24 h treatment. Data are presented as mean ± SEM of five independent experiments

The analysis of the viability of PBL following MG treatment for 24 h allowed to obtain an IC_50_ value of 761 μg/mL, which demonstrated a cytotoxicity lower than that manifested on the two leukaemia cell lines (Fig. 9).

## Discussion

Great attention has been dedicated to the potential of many extracts and naturally occurring compounds in the prevention/counteraction of chronic diseases [28–30]. Numerous studies have demonstrated that molecules that exhibit antioxidant properties such as polyphenols, isothiocyanates and other compounds counteract cardiovascular, neurodegenerative diseases and the carcinogenic process at various levels by acting through multiple mechanisms [31–34]. Extracts from plant origin may contain various compounds with useful biological properties, thus configuring themselves as complex mixtures with multi-target activities, capable of inhibiting or modulating simultaneously numerous critical targets [25, 35, 36].

Edible mushrooms, including MG, possess antioxidant, antimicrobial and anti-inflammatory properties [7, 37–39], suggesting their potential application as chemopreventive agents. The purpose of this work was, therefore, to evaluate whether MG extract is able, thanks to its antioxidant activity to exhibit chemopreventive activities. The research focused on the evaluation of numerous in vitro end-points in two leukemic cell lines (Jurkat and HL-60 cells) and subsequently in healthy lymphocytes (PBL).

Among scientific literature, strong discrepancies can be observed when comparing TP and TF content of MG extracts., These differences are probably due to extraction methods, mushroom provenance and the fact that TP and TF might be expressed in different ways. Papers reporting the TP of wild edible mushrooms other than MG differ in a very wide range in the order of mg/mL [6, 40–42].

HPLC-MS/MS analysis of MG ethanolic extract resulted in quantification of four phenolic acids, giving the total sum of 26.66 μg/g. Dominant phenolic acid was *p*-hydroxybenzoic acid, in accordance with previously published data on MG [6]. All the differences that can be observed when comparing the previously published data for MG and other wild mushrooms should be attributed to the changes that occur during the mushrooms harvest and postharvest period of time, due to the enzymatic and oxidative processes [43, 44].

It has been shown that the strong antiradical activity of mushroom extracts on different radical species is due to the presence of different phenolic compounds and flavonoids [45, 46].

Determination of DPPH^**·**^ scavenging capacity is a commonly employed assay in antioxidant studies, Ferreira et al. [47] analysed DPPH^**·**^ scavenging capacity of different mushroom methanolic extracts finding that DPPH EC_50_ was in the range of 8–50 mg/mL. Similarly, Puttaraju et al. [48] analysed methanolic and water extracts of 23 different edible mushrooms and found that DPPH EC_50_ values were in the same order of magnitude.

Only few studies have previously evaluated MG extracts antioxidant activity. Our data show MG ethanolic extract ability to scavenge different radicals such as DPPH^**·**^, O_2_^·-^, OH^·^, NO and to exert antioxidant activity by reducing Fe^3+^ ions (FRAP). These data are in agreement with those previously published [5, 6, 8] which found that MG water, ethanolic, methanolic and chloroformic extracts exert DPPH^**·**^ and OH^·^ radical scavenging activity with EC_50_ values in the order of μg/mL. Similarly, FRAP result is in agreement with previous report [6]. All together, these data suggest that MG extract is characterized by a strong radical scavenging activity mainly related to its phenols content as previously reported by Karaman et al. [6].

Due to the strong antioxidant activity, it was possible to hypothesize a chemopreventive role for MG ethanolic extract.

A chemopreventive agent can act in different ways: by modulating the biotransformation enzymes involved in the activation/detoxification processes of carcinogens or by stimulating apoptosis and inhibiting the proliferation of transformed cells [49].

Even though some studies reporting pro-apoptotic and anti-proliferative effects of mushroom extract have already been published [50, 51], to our knowledge no data on MG effects in leukemic cell lines are available. Therefore, specific mechanisms of cell death, apoptosis and/or necrosis and MG ability to modulate cell-cycle were investigated.

Results show that MG is able to significantly induce apoptosis in a dose- and time- related manner in both leukemic cell lines. Moreover, it does not modulate, in any way, Jurkat cell-cycle while it inhibits HL-60 proliferation by causing a slowdown in G_2_ /M phase after 24 h treatment, which results in a real block after 48 h in G_0_/G_1_ phase with a corresponding decrease in the percentage of cells in phase S and G_2_/M. This effect is confirmed after 72 h of treatment. These data are in agreement with previous reports showing that different flavonoids and polyphenols exert cell-cycle arrest in HL-60 [52]. A possible hypothesis to explain the differences between MG treatment effect on Jurkat and HL-60 cell cycle resides in the substantial differences that exist between these two cell lines. Jurkat cells are a lymphocyte cell line in an advanced state of maturation and differentiation, while HL-60 are a highly undifferentiated immature promyelocytic cell line. Future studies are needed to clarify whether these differences are responsible for the different effect of MG on the cell cycle [53].

In order to elucidate which mechanism is responsible for the pro-apoptotic effect we evaluated the expression level of genes such as FAS, BAX and BCL2.

FAS receptor belongs the family of death receptors, it is located on the cell membrane and the binding to its ligand leads to apoptosis through caspase-8 activation which directly activates caspase 3 and simultaneously promote Bid cleavage leading to mitochondrial membrane potential loss [54]. Moreover, it has been demonstrated that FAS over-expression induces apoptosis in Jurkat cell and other malignant T-cell lines [55]. In our study, 16 h treatment with MG induced FAS mRNA expression in both Jurkat and HL-60 cells. Bax and Bcl-2 are mitochondrial proteins, while Bax exhibits pro-apoptotic activity, Bcl-2 is considered an anti-apoptotic and is often overexpressed in different cancers [56, 57]. In order to induce apoptosis in cancer cells, most therapies are based on stimulating the expression of Bax and/or suppressing Bcl-2 protein. In this study we contemporaneously observed, in Jurkat cells, an increase of BAX and a decrease of BCL2 gene expression level after 250 μg/mL MG treatment. Otherwise, in HL-60 cells a reduction of BCL2 level was found, while BAX was not affected. Our data demonstrate that BAX/BCL2 mRNA expression ratio significantly increased in both cell lines suggesting the involvement of these two proteins in the progression of the apoptotic cascade induced by MG.

It is known, that high ROS intracellular level is a common characteristic of leukaemic and other cancer cells [58]. This feature has been observed in numerous leukaemic cell lines and also in cells from patients with different types of leukaemia [59]. Therefore, it is generally accepted that increased ROS production is important for the proliferation of hematological malignancies [60, 61]. Due to ROS importance in sustaining leukemic cell proliferation and survival, the reduction of their levels could represent an effective strategy to reduce leukemic cells proliferation [59]. Moreover, Aronis et al. [62] demonstrated that a reduced ROS level could induce apoptosis by the involvement of death receptor in Jurkat cells.

In this context, our data show MG ability to exert scavenging and antioxidant activity leading to a significant reduction of ROS intracellular level which contributes to explain the pro-apoptotic effect induced by the activation of the extrinsic apoptotic pathway as indicated by FAS increased expression level.

**Fig. 10:**
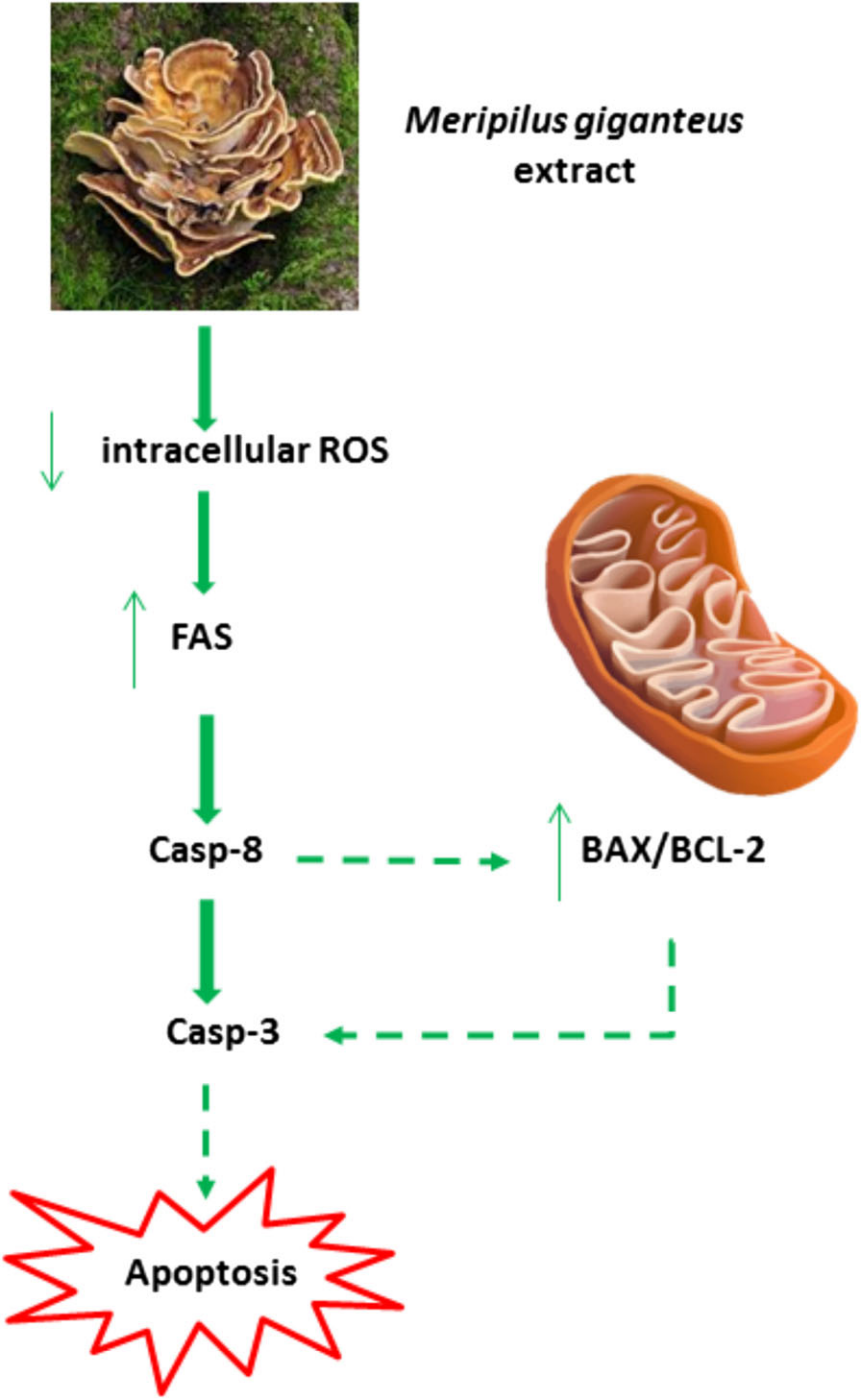
Hypothesised pro-apoptotic mechanism of MG extract in leukemic cell lines. Due to its phenolic content and antioxidant activity, MG ethanolic extract induces a decrease of ROS intracellular level. In leukemic cell, ROS decrease has been related to FAS recruitment leading to the activation of the extrinsic apoptotic pathway. A possible explanation of the observed increase of BAX/BCL-2 ratio consists in the fact that FAS ligands lead to apoptosis through caspase-8 activation which on one hand promote the extrinsic apoptotic pathway, while on the other promote mitochondrial membrane potential loss through BAX/BCL2 ratio increase

The hypothesised pro-apoptotic mechanism of MG extract is summarized in the scheme shown in Fig. 10.

A fundamental feature of a good chemopreventive agent is the low toxicity towards healthy cells and the relative selectivity of action against tumor cells [63]. Therefore, the study was completed by evaluating MG cytotoxicity on PBL after 24 h of treatment.

MG demonstrated good selectivity as shown by the IC_50_, calculated for PBL by interpolation of the dose response curve, that resulted 2 and 1.7 times higher than those obtained on Jurkat and HL-60 cells respectively.

## Conclusions

MG demonstrated good pro-apoptotic capacity in both Jurkat and HL-60 cell lines, with a predominant antiproliferative effect in HL-60 and to be partially selective towards leukemic cells. These findings allow to propose MG extracts as possible candidate as chemopreventive agents.

## Abbreviations

7-AAD: 7-aminoactinomycin D
AAE/g: mg ascorbic acid equivalents
DCF: 2′,7′-dichlorofluorescein
DCFH-DA: 2′-7′-dichlorodihydrofluorescin diacetate
DMSO: Dimethyl sulfoxide
DPPH^·^: DPPH radical
EtOH: Ethanol
FBS: Fetal bovine serum
FCM: Flow cytometry
Fe^2+^: Ferrous ions
FRAP: Ferric reducing antioxidant power
GAE/g: mg gallic acid equivalents
HL-60 cells: Acute promyelocytic leukemia
Jurkat cells: Acute T-cell lymphoblastic leukemia
L-GLU: L-Glutamine
MG: *Meripilus giganteus*
MGPS: MG a polysaccharide
MRM mode: Multiple reaction monitoring mode
NBT: Nitro blue tetrazolium
NEDA: N-(1-naphthyl) ethylenediamine dihydrochloride
NO^·^: Nitric oxide
O_2_^·-^: Superoxide anion radical
OH^·^: Hydroxyl radical
PBL: Human peripheral blood lymphocytes
PHA: Phitohemagglutinin
PI: Propidium iodide
PMS: 5-methylphenazin-5-ium methyl sulfate
PS: Penicillin-strptomicin
QE/g: mg quercetin equivalents
RPMI 1640 medium: roswell park memorial institute
SNP: Sodium nitroprusside
TBA: Tiobarbituric acid
TCA: Trichloroacetic acid
TF: Total flavonoid
TP: Total phenolic content
TPTZ: 2,4,6-tris(2-pyridyl)-s-triazine
